# Structural basis for broad substrate specificity of UDP-glucose 4-epimerase in the human milk oligosaccharide catabolic pathway of *Bifidobacterium longum*

**DOI:** 10.1038/s41598-019-47591-w

**Published:** 2019-07-31

**Authors:** Young-Woo Nam, Mamoru Nishimoto, Takatoshi Arakawa, Motomitsu Kitaoka, Shinya Fushinobu

**Affiliations:** 1 Department of Biotechnology, The University of Tokyo, Tokyo, 113-8657 Japan; 2 Food Research Institute, National Agriculture and Food Research Organization, Tsukuba, Ibaraki 305-8642 Japan; 3 Collaborative Research Institute for Innovative Microbiology, The University of Tokyo, Tokyo, 113-8657 Japan; 4 Faculty of Agriculture, Niigata University, Niigata, 950-2181 Japan; 5 Present Address: Department of Biomedical and Pharmaceutical Sciences & Structural Biology Research Center, Chapman University School of Pharmacy, Irvine, CA 92618 USA

**Keywords:** Enzyme mechanisms, Bacteria, X-ray crystallography

## Abstract

Infant gut-associated bifidobacteria has a metabolic pathway that specifically utilizes lacto-*N*-biose I (Gal-β1,3-GlcNAc) and galacto-*N*-biose (Gal-β1,3-GalNAc) from human milk and mucin glycans. UDP-glucose 4-epimerase (GalE) from *Bifidobacterium longum* (bGalE) catalyzes epimerization reactions of UDP-Gal into UDP-Glc and UDP-GalNAc into UDP-GlcNAc with the same level of activity that is required to send *galacto-*hexoses into glycolysis. Here, we determined the crystal structures of bGalE in three ternary complex forms: NAD^+^/UDP, NAD^+^/UDP-GlcNAc, and NAD^+^/UDP-Glc. The broad specificity of bGalE was explained by structural features of the binding pocket for the *N*-acetyl or C2 hydroxy group of the substrate. Asn200 is located in a pocket of the C2 group, and its side chain adopts different conformations in the complex structures with UDP-Glc and UDP-GlcNAc. On the other side, Cys299 forms a large pocket for the C5 sugar ring atom. The flexible C2 pocket and the large C5 pocket of bGalE are suitable for accommodating both the hydroxy and *N*-acetyl groups of the substrate during sugar ring rotation in the catalytic cycle. The substrate specificity and active site structure of bGalE were distinct from those of *Esherichia coli* GalE but similar to those of human GalE.

## Introduction

Bifidobacteria are anaerobic gram-positive bacteria belonging to the genus *Bifidobacterium* and are usually found in the gastrointestinal tract of humans and animals^1^. It is known that they play a significant role in human health by acting with various transporters, glycosidases, and metabolic enzymes^2,3^. Several species of *Bifidobacterium*, e.g., *Bifidobacterium longum* subsp*. longum* (*B. longum*), *Bifidobacterium bifidum*, *Bifidobacterium breve*, and *Bifidobacterium longum* subsp. *infantis*, are the predominant intestinal bacteria in breastfed infants^4–6^. Recent studies revealed that the infant gut-associated bifidobacteria has specialized metabolic pathways for utilizing oligosaccharides contained in human milk with a considerably large amount (up to 12–24 g/L), suggesting a symbiotic relationship between humans and these microorganisms^7–9^. The human milk oligosaccharides (HMOs) consist of more than 240 different kinds of molecular species with a degree of polymerization of more than three^10^. The structural unit of lacto-*N*-biose I (Gal-β1,3-GlcNAc, LNB) is predominantly present in HMOs but not in milk of other mammals^11^, and LNB has been shown to be the bifidus factor, which promotes growth of infant gut-associated bifidobacteria^12,13^. The metabolic pathway dedicated for utilization of LNB also utilizes galacto-*N*-biose (Gal-β1,3-GalNAc, GNB) that is present in glycoconjugates, such as mucin glycoproteins^14,15^. The gene cluster for the GNB/LNB pathway in bifidobacteria is composed of a specific ABC transporter and four intracellular enzymes, GNB/LNB phosphorylase (GLNBP, EC 2.4.1.211; locus tag = BLLJ_1623), *N*-acetylhexosamine 1-kinase (NahK, EC 2.7.1.162; BLLJ_1622), UDP-glucose—hexose-1-phosphate uridylyltransferase (GalT, EC 2.7.7.12; BLLJ_1621), and UDP-glucose 4-epimerase (GalE, EC 5.1.3.2; BLLJ_1620). Of note, here we use the accepted name of GalE recommended by Nomenclature Committee of IUBMB-IUPAC, although the other name “UDP-galactose 4-epimerase” has been generally used in the structural biology community until recently. We previously reported crystal structures of the solute-binding protein of the GNB/LNB-specific transporter^16^, GLNBP^17^, and NahK^18^. Here, we focused on structural features of GalE from *B. longum* JCM1217 (bGalE).

**Figure 1:**
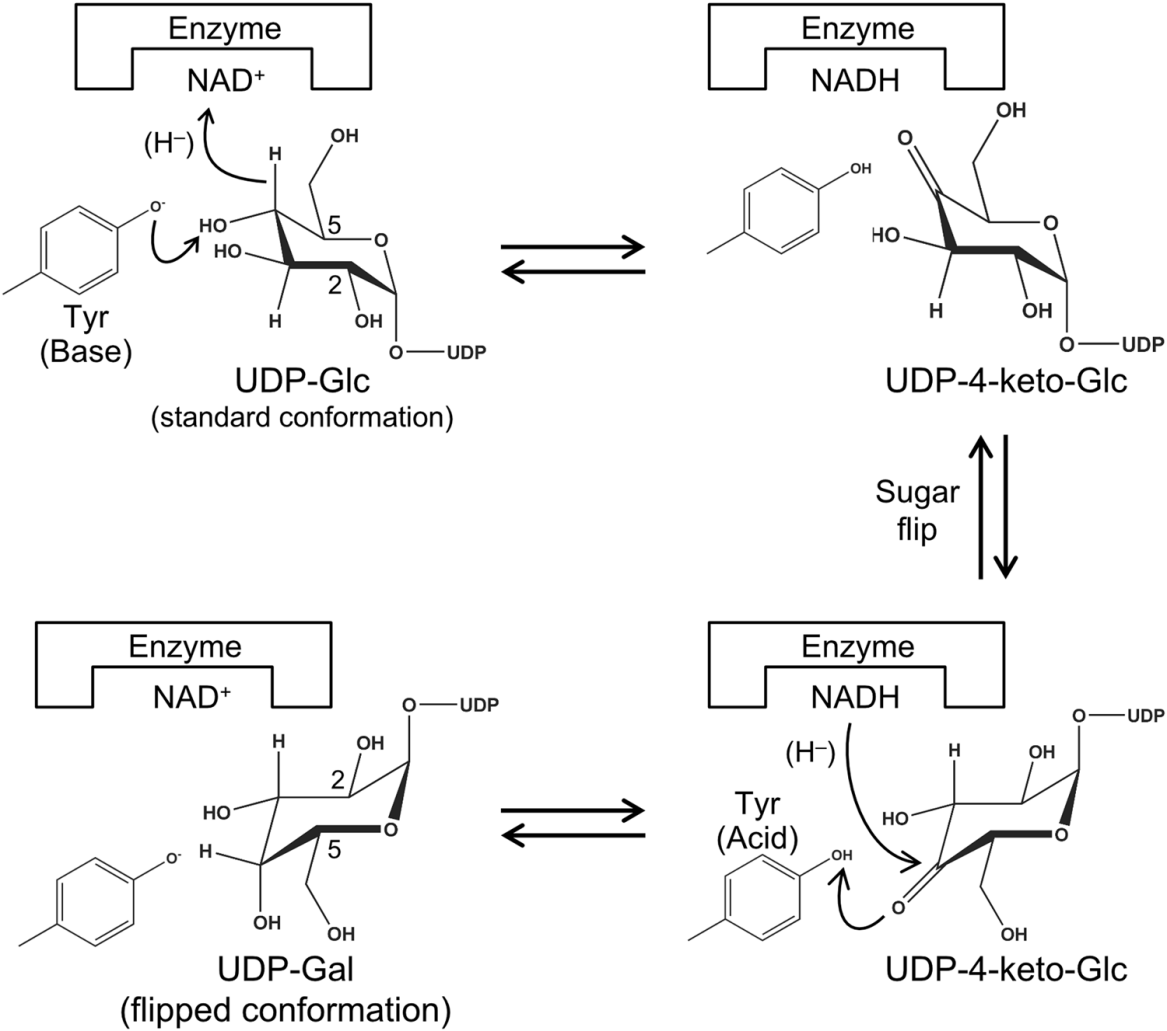
Proposed reaction mechanism of GalE. The catalytic base/acid tyrosine residue of bGalE is Tyr150. The NAD^+^ cofactor is tightly bound to the protein as a prosthetic group and facilitates catalysis via hydride transfer.

**Figure 2:**
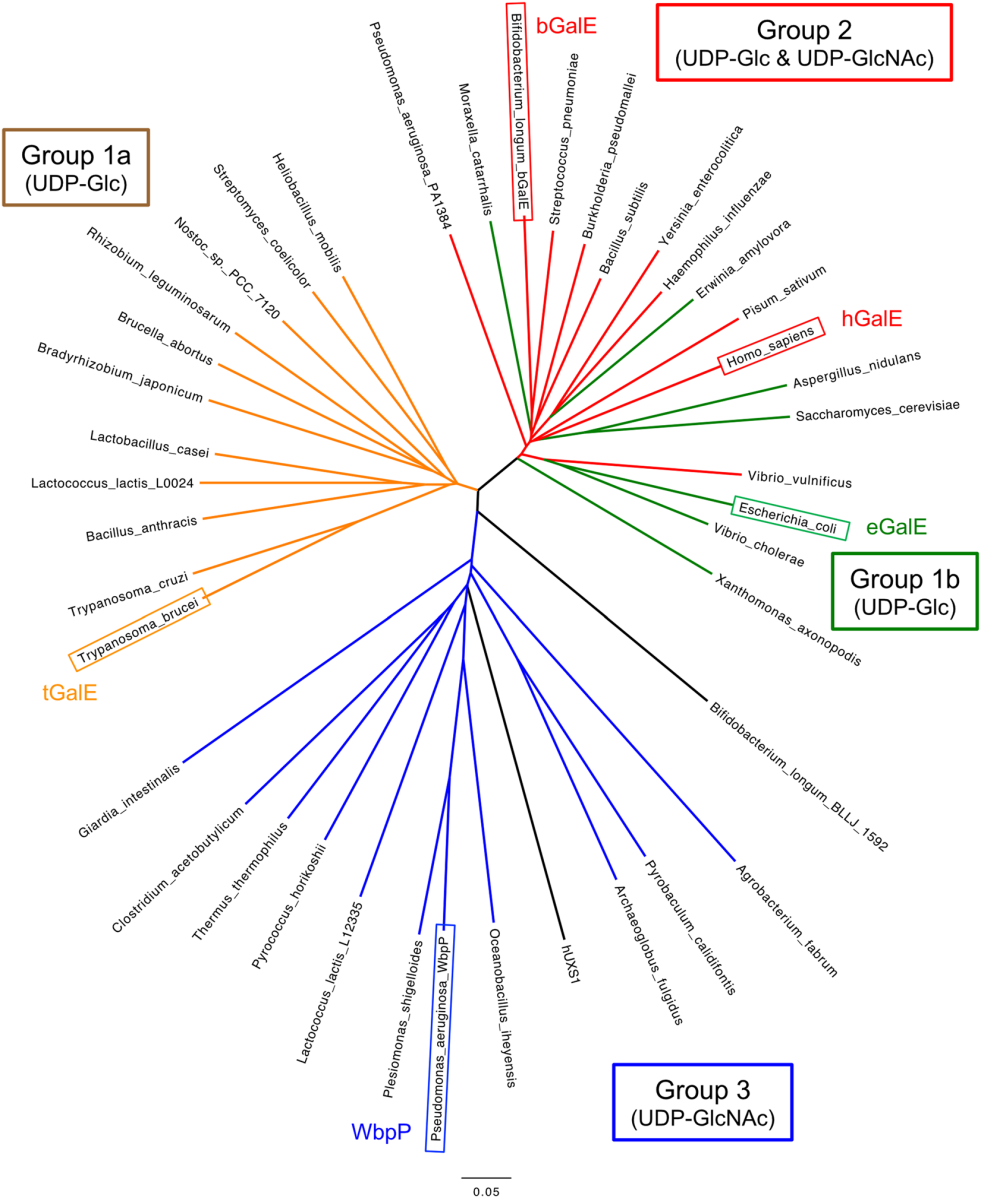
Phylogenetic tree of GalEs and UDP-xylose synthase. Source organisms of biochemically and structurally characterized GalE enzymes and those of uncharacterized protein sequences are shown. The group classification and substrate specificity prediction are performed according to the method described in Ishiyama *et al*.^32^. Human UDP-xylose synthase 1 (hUXS1) is also shown. Bar, 5% sequence divergence.

GalE catalyzes NAD^+^-dependent oxidoreductive interconversion of *gluco*- and *galacto*-hexoses (C4-epimerization) linked to UDP (Fig. 1) and generally plays a key role in the metabolism of galactose in various organisms^19–21^. Crystal structures of GalEs from *Escherichia coli* (eGalE)^22–27^, *Trypanosoma brucei* (tGalE)^28^, human (hGalE)^29–31^, *Pseudomonas aeruginosa* (WbpP)^32^, *Pyrobaculum calidifontis*^33^, *Aspergillus nidulans*^34^, and several others have been reported. Ishiyama *et al*. classified GalE enzymes into three major groups based on their substrate specificity^32^. Group 1 enzymes preferentially catalyze the epimerization between UDP-Glc and UDP-Gal (eGalE and tGalE), group 2 enzymes do not show a preference for either UDP-Glc/UDP-Gal or UDP-GlcNAc/UDP-GalNAc (hGalE), and group 3 enzymes preferentially catalyze the epimerization between UDP-GlcNAc and UDP-GalNAc (WbpP) (Table 1). Group 1 enzymes are further divided into two subfamilies (1a and 1b) due to their separation in the phylogenetic tree (Fig. 2). bGalE catalyzes epimerization reactions of UDP-Gal into UDP-Glc and UDP-GalNAc into UDP-GlcNAc with the same level of activity and is categorized into group 2^15^. Amino acid sequence identities between bGalE and the representative of each group are as follows: hGalE (group 2) = 54.0%, eGalE (group 1b) = 54.3%, tGalE (group 1a) = 37.5%, and WbpP (group 3) = 24.3%. GalEs are classified as SDR1E family of a large short-chain dehydrogenases/reductase (SDR) superfamily, which includes oxidoreductases, epimerases, and lyases^35^. In this study, we describe the crystal structures of bGalE and compare them with GalEs from other organisms.

**Table 1:** Enzyme activities of GalE enzymes.

	UDP-Glc	UDP-Gal	UDP-GlcNAc	UDP-GalNAc	Note	*Ref*.
bGalE	—	158	147	—	Specific activity (U/mg) at 1 mM UDP-sugar	^15^
hGalE	—	33.8	—	26	Specific activity (U/mg) at 0.41 mM UDP-Gal or 0.66 mM UDP-GalNAc	^27^
hGalE (C307Y)		1.21		0.02	Relative activity compared with the wild type enzyme at 2 mM UDP-Gal or 1.89 mM UDP-GalNAc	^43^
eGalE	—	23.9	—	0.003	Specific activity (U/mg) at 0.41 mM UDP-Gal or 0.66 mM UDP-GalNAc	^27^
eGalE (Y299C)	—	5.1	—	0.69		
tGalE	—	5.9	—	—	Specific activity (U/mg) at 20 mM UDP-Gal	^54^
WbpP	0.124	0.188	120	271	*k*_cat_ (min^−1^). *K*_m_ values were similar for all substrates (197~251 uM)	^55^

—, Not determined.

## Results and Discussion

### Overall structure

**Figure 3:**
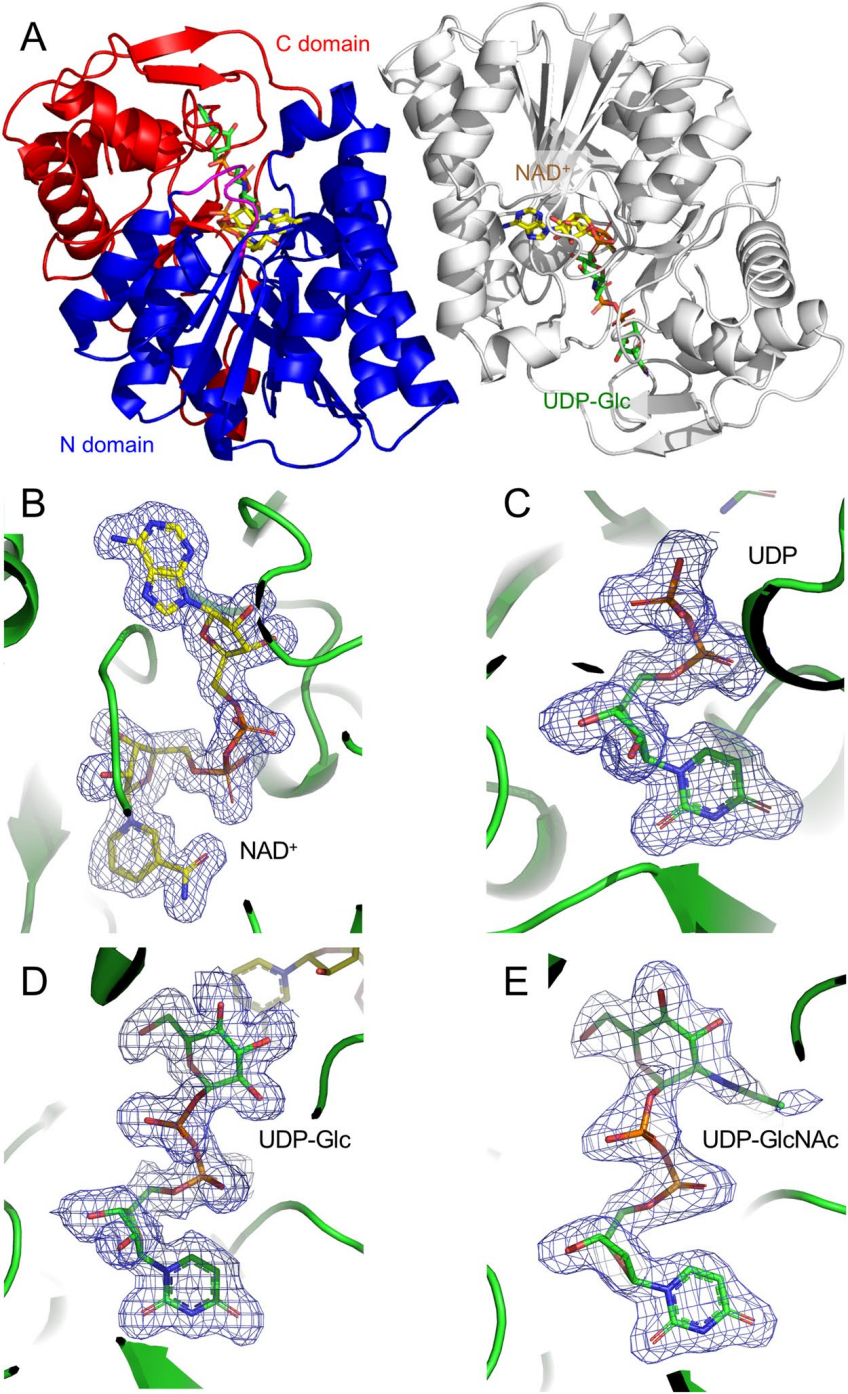
Crystal structure of bGalE. (**A**) Overall structure of the biological dimer assembly. One protomer is shown in blue (N domain, residues 1–177 and 236–262) and red (C domain, residues 178–235 and 263–340), and the other symmetry-related protomer is shown in gray. *m*F_o_-*D*F_c_ omit map of NAD^+^ (**B**) and UDP (**C**) in the NAD^+^ + UDP complex, UDP-Glc (**D**), and UDP-GlcNAc (**E**) are shown with a contour level of 3.0σ. NAD^+^ and UDP or UDP-sugar molecules are shown with yellow and green sticks, respectively.

The molecular masses of the purified recombinant bGalE protein (348 amino acids) as deduced from the amino acid sequence, estimated by SDS-PAGE and gel filtration chromatography were 38.3, 38, and 68 kDa, respectively, suggesting that this protein is dimeric in solution. Crystals of bGalE belong to the *P*6_5_22 space group and contain one monomer per asymmetric unit. Since the recombinant bGalE protein contains one NAD^+^ molecule per monomer as a noncovalently bound prosthetic group, we prepared ternary complex form crystals with UDP, UDP-Glc, or UDP-GlcNAc by cocrystallization and determined their crystal structures at 1.8–2.0 Å resolution (Table 2). A plausible dimer assembly formed by a crystallographic 2-fold rotation symmetry is present in the crystal packing (Fig. 3A). A molecular interface analysis using PISA^36^ indicates that the whole surface area, buried area, and *ΔG*^int^ value (the solvation free energy gain upon formation of the assembly) are 23,790 Å^2^, 6,340 Å^2^, and –44.7 kcal/mol, respectively. The dimer interface consists of a bundle of 4 α-helices and contains 8 hydrogen bonds and 4 salt bridges. bGalE has a typical fold of SDR superfamily^35^. The overall monomer protein structure can be roughly divided into an N-terminal NAD^+^ binding domain (N domain, residues 1–177 and 236–262) and a C-terminal UDP-sugar binding domain (C domain, residues 178–235 and 263–340). The N domain adopts a typical Rossmann fold composed of a seven-stranded parallel β-sheet flanked by nine α-helices. The C domain possesses an α/β motif consisting of four α-helices and four β-strands. The three ternary complex structures have virtually the same main chain structures because the root mean square deviation (RMSD) values for the Cα atoms (no distance cutoff) between them are less than 0.18 Å. The main chain structure of bGalE is similar to those of bacterial and eukaryotic GalEs. Cα RMSD values (distance cutoff = 2.0 Å) of the UDP-GlcNAc complex with hGalE (PDB ID 1HZJ, chain B), eGalE (PDB ID 1XEL), tGalE (PDB iD 2CNB, chain A), and WbpP (PDB ID 1SB8) are 0.69 Å (306 a.a.), 0.88 Å (284 a.a.), 0.72 Å (239 a.a.), and 1.41 Å (231 a.a.), respectively.

**Table 2:** Data collection and refinement statistics.

	UDP + NAD^+^	UDP-GlcNAc + NAD^+^	UDP-Glc + NAD^+^
**Data collection**^**a**^			
Beamline	BL17A	BL5A	NW12A
Wavelength	0.979	1.000	1.000
Space group	*P*6_5_22	*P*6_5_22	*P*6_5_22
Unit cell (Å)	*a* = *b* = 70.1, *c* = 322.2	*a* = *b* = 69.4, *c* = 322.0	*a* = *b* = 70.4, *c* = 322.6
Resolution (Å)^a^	48.50–1.80 (1.84–1.80)	48.13–2.00 (2.05–2.00)	48.62–1.80 (1.84–1.80)
Total reflections	952,581 (55,646)	551,727 (41,132)	800,942 (45,652)
Unique reflections	45,059 (2,591)	32,430 (2,323)	44,487 (2,504)
Completeness^b^	100.0 (100.0)	100.0 (100.0)	98.8 (97.7)
Multiplicity^b^	21.1 (21.5)	17.0 (17.7)	18.0 (18.2)
Mean *I*/*σ*(*I*)^b^	9.3 (4.0)	18.1 (3.7)	24.9 (6.9)
*R*_merge_^b^	0.253 (0.947)	0.133 (0.855)	0.100 (0.552)
CC_1/2_^b^	0.983 (0.961)	0.993 (0.974)	0.999 (0.974)
**Refinement**^**c**^			
Resolution	40.17–1.80	34.75–2.00	36.78–1.80
No. of reflections	42,417	30,845	42,233
*R* factor/*R*_free_ (%)	14.9 (18.0)	18.1 (22.1)	16.1 (19.0)
No. of atoms	3,033	2,898	2,990
No. of solvents	1 (UDP), 1 (NAD^+^), 1 (Mg^2+^), 375 (water)	1 (UDP-GlcNAc), 1 (NAD^+^), 2 (Mg^2+^), 211 (water)	1 (UDP-Glc), 1 (NAD^+^), 1 (Mg^2+^), 322 (water)
RMSD from ideal values			
Bond lengths (Å)	0.014	0.012	0.015
Bond angles (°)	1.73	1.66	1.77
Ramachandran plot (%)			
Favored	99.1	98.8	99.1
Allowed	0.6	0.9	0.6
Outlier	0.3	0.3	0.3
PDB ID	6K0G	6K0H	6K0I

^a^Calculated using XDS.

^b^Values in parentheses are for the highest resolution shell.

^c^The structures were refined using diffraction datasets processed by HKL2000.

### NAD^+^ binding site

**Figure 4:**
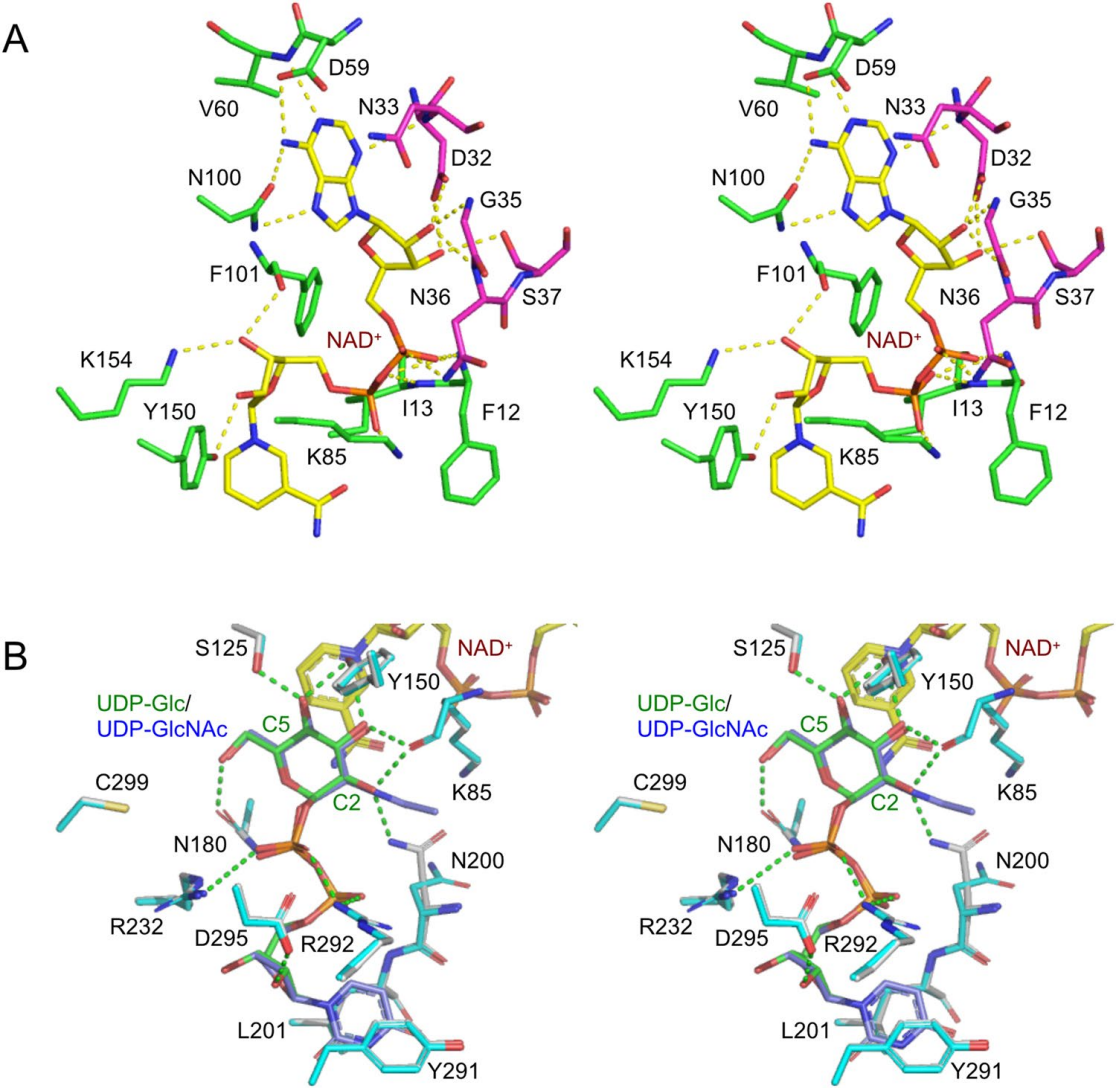
Stereoview of the cofactor and substrate binding sites of bGalE. (**A**) NAD^+^ binding site of the UDP complex structure. (**B**) Superimposition of the complex structures with UDP-Glc (white and green for protein and UDP-Glc) and UDP-GlcNAc (cyan and blue for protein and UDP-GlcNAc). NAD^+^ and hydrogen bonds are shown as yellow sticks and dotted lines, respectively.

The electron density of the NAD^+^ molecule bound in the N domain was clearly observed in all three complex structures. Figure 3B shows the *m*F_o_-*D*F_c_ omit map of NAD^+^ in the UDP complex. The nicotinamide ribose and adenine ribose moieties are in *syn*-C2′-endo and *anti*-C2′-endo conformations, respectively. The ribose conformations of NAD^+^ are identical to those observed in eGalE^37^ and other GalE enzymes studied so far. Figure 4A shows interactions of the NAD^+^ molecule with bGalE. Residues recognizing the NAD^+^ cofactor are basically conserved in GalEs. In bGalE, the nicotinamide ribose moiety interacts with Phe101, Lys154, and Tyr150, the pyrophosphate moiety interacts with Phe12, Ile13, and Lys85, and the adenosine base moiety interacts with Asn33, Asp59, Val60, and Asn100. A loop region (Asp32–Ser37, magenta in Fig. 4A) forms several direct hydrogen bonds with the base, ribose, and phosphate groups of the adenosine moiety.

**Figure 5:**
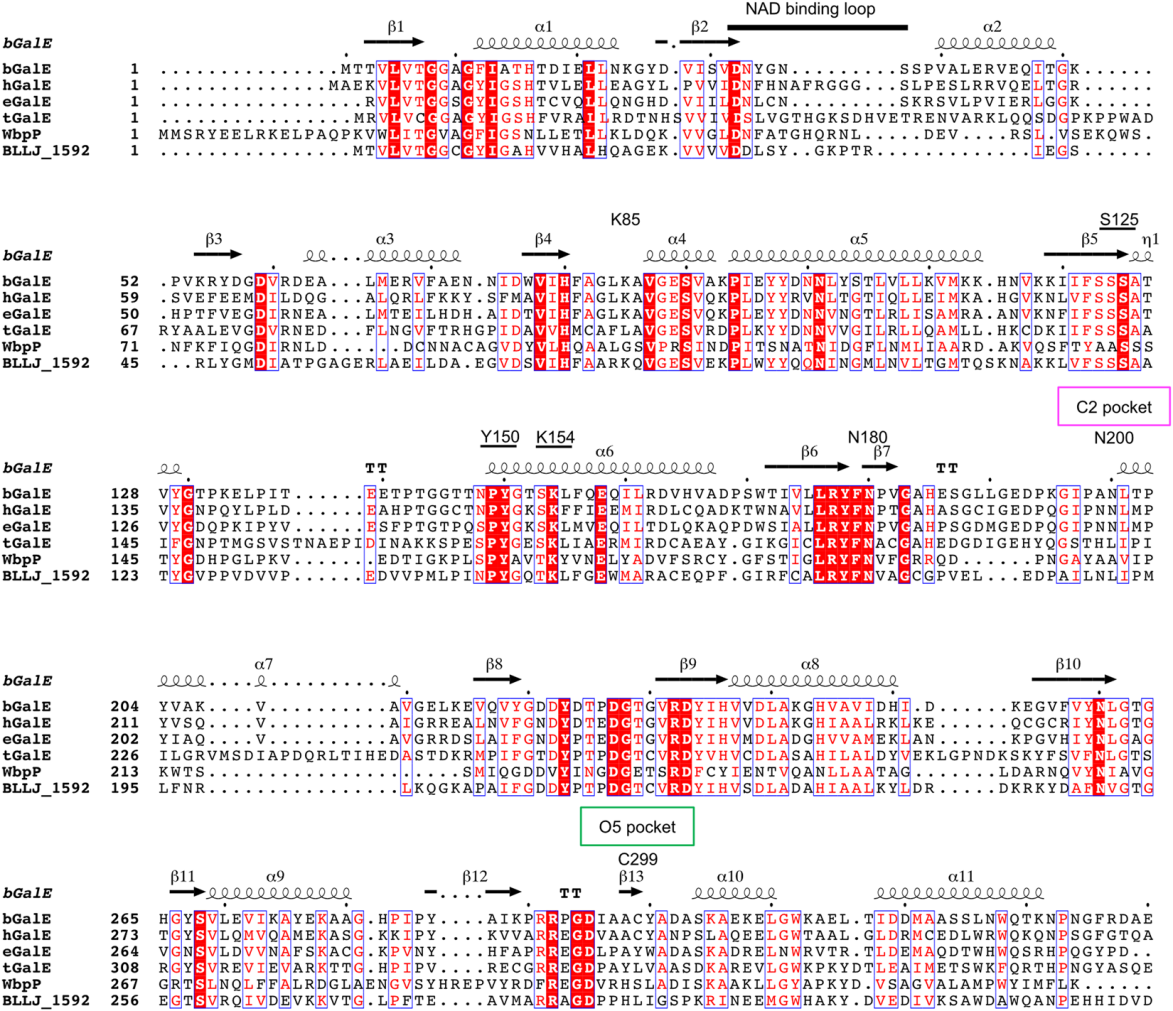
Amino acid sequence alignment of GalEs. Representatives of group 2 (bGalE and hGalE), group 1b (eGalE), group 1a (tGalE), group 3 (WbpP), and a paralog of bGalE (BLLJ_1592) are shown. The catalytic triad residues are underlined.

The cofactor NAD^+^ was intrinsically bound to bGalE without any supplementation throughout experimental processes in all complexes. It was reported that when NAD^+^ was removed from the GalE proteins, the proteins were irreversibly denatured^37^. The loop region of Asp32–Ser37 in the N domain interacts with the adenosine moiety of NAD^+^ (magenta in Fig. 3A). The corresponding region (residues 32–43) in *P. calidifontis* GalE was defined as the “NAD-binding loop”^33^. The NAD-binding loop is present in all GalE homologs (Fig. 5). In contrast, l-threonine dehydrogenases (l-ThrDHs), which share similar structural fold with GalEs, lack this loop and easily release NAD^+^ from the protein^38^. Sakuraba *et al*. indicated that the NAD-binding loop of GalEs plays a key role in preventing the release of the catalytically relevant cofactor and contributing to the protein stability of *P. calidifontis* GalE^33^.

### UDP-sugar binding site

As shown in Fig. 3C–E, the electron densities of UDP, UDP-Glc, and UDP-GlcNAc were clearly observed. Interactions of UDP-Glc and UDP-GlcNAc with bGalE are shown in Fig. 4B. Residues recognizing the UDP moiety are basically conserved in GalEs. In bGalE, the UDP moiety is recognized by stacking and hydrophobic interactions by Tyr291 and Leu201, hydrogen bonds by Asp295 and Asn180, and salt bridges by Arg292 and Arg232. These interactions are identically present in the UDP complex (data not shown). The hydroxy groups of the Glc moiety are recognized by hydrogen bonds from the main chain of Lys85 (O2 and O3) and the side chain atoms of Asn200 (O2), Tyr150 (O3 and O4), Ser125 (O4), and Asn180 (O6). The GlcNAc moiety is similarly recognized, but a significant displacement of the side chain of Asn200 was observed. In the UDP-GlcNAc complex, the side chain of Asn200 adopts a distinct conformation (designated as “swing out”) from that in the UDP-Glc complex (“swing in”) to accommodate the 2-*N*-acetyl group. This phenomenon was also reported for the corresponding residue (Asn207) in the UDP-GlcNAc complex of hGalE^30^ (discussed below).

The glucopyranose rings of the UDP-sugars in bGalE are both in the “standard” conformation (Fig. 1), in which the H4 atom faces the pro-*S* side of the NAD^+^ nicotinamide. The distances between the C4 atom of the sugar ring and the C4 atom of the nicotinamide ring are 3.4 Å and 3.5 Å in the UDP-Glc and UDP-GlcNAc complex structures, respectively. In the proposed reaction mechanism of GalE, a hydride transfer is thought to occur between these carbon atoms, leading to formation of a 4-ketopyranose intermediate and NADH (Fig. 1)^37^. It has been reported so far that the distance between the C4–C4 atoms in a productive mode is within the range of 3.0 ~ 3.7 Å^22,26,29,30,34,39^. Tyr150 forms a direct hydrogen bond (2.6 Å) with the O4 atom in both the UDP-Glc and UDP-GlcNAc complex structures. The corresponding residue of hGalE (Tyr157) is the catalytic base residue (proton acceptor) from the O4 hydroxy in the first reaction step (Fig. 1)^29^. In the subsequent reaction steps, the sugar moiety of the substrate is assumed to flip its sugar ring, and the nicotinamide ring of the reduced cofactor (NADH) transfers back the hydride from the *si*-face to the opposite face of the C4-keto-intermediate to produce an epimer^19^. In the latter step, the catalytic tyrosine residue acts as a general acid (proton donor) to the O4 hydroxy group of the sugar moiety. A comprehensive study using X-ray crystallography, *in situ* NMR, and steady-state and stopped-flow kinetics revealed a detailed reaction mechanism of human UDP-xylose synthase 1 (hUXS1; EC 4.1.1.35; other name: UDP-glucuronic acid decarboxylase), which also belongs to the SDR1E family of SDR superfamily^40^. In addition to the decarboxylation reaction that forms xylose from glucuronic acid, the proposed reaction mechanism involves base/acid catalysis by Tyr147, which is located at the corresponding position (near the C4 atom of the sugar moiety) to Tyr150 of bGalE.

A completely conserved SYK catalytic triad motif has been recognized in SDR superfamily enzymes^35^. In bGalE, Ser125, Tyr150, and Lys154 correspond to the triad (Fig. 5). Lys154 contributes to the binding of nicotinamide ribose, while Ser125 and Tyr150 form direct hydrogen bonds with the O3 and O4 hydroxy groups of the Glc or GlcNAc (Fig. 4), respectively. These residues appear to support precise transfer of hydride and proton, which is the most important step of GalE catalysis. For eGalE, site-directed mutagenesis studies indicated the catalytic importance of the triad residues (Ser124, Tyr149, and Lys153)^41,42^. Structural analyses on mutants of the Ser and Tyr residues were also reported^24,26^. Tyr149 and Ser124 are in position to participate in the acid-base catalysis required to drive hydride transfer and synergistically contribute to the catalysis by coordinating the reactive O4 hydroxy group^20^. Asn180, which recognizes the O6 hydroxy of the sugar moiety, is also completely conserved (Fig. 5). The other three residues involved in forming the sugar binding pocket (Lys85, Asn200, and Cys299) exhibit variety in the three GalE groups (1, 2, and 3) and prescribe their substrate specificity.

**Figure 6:**
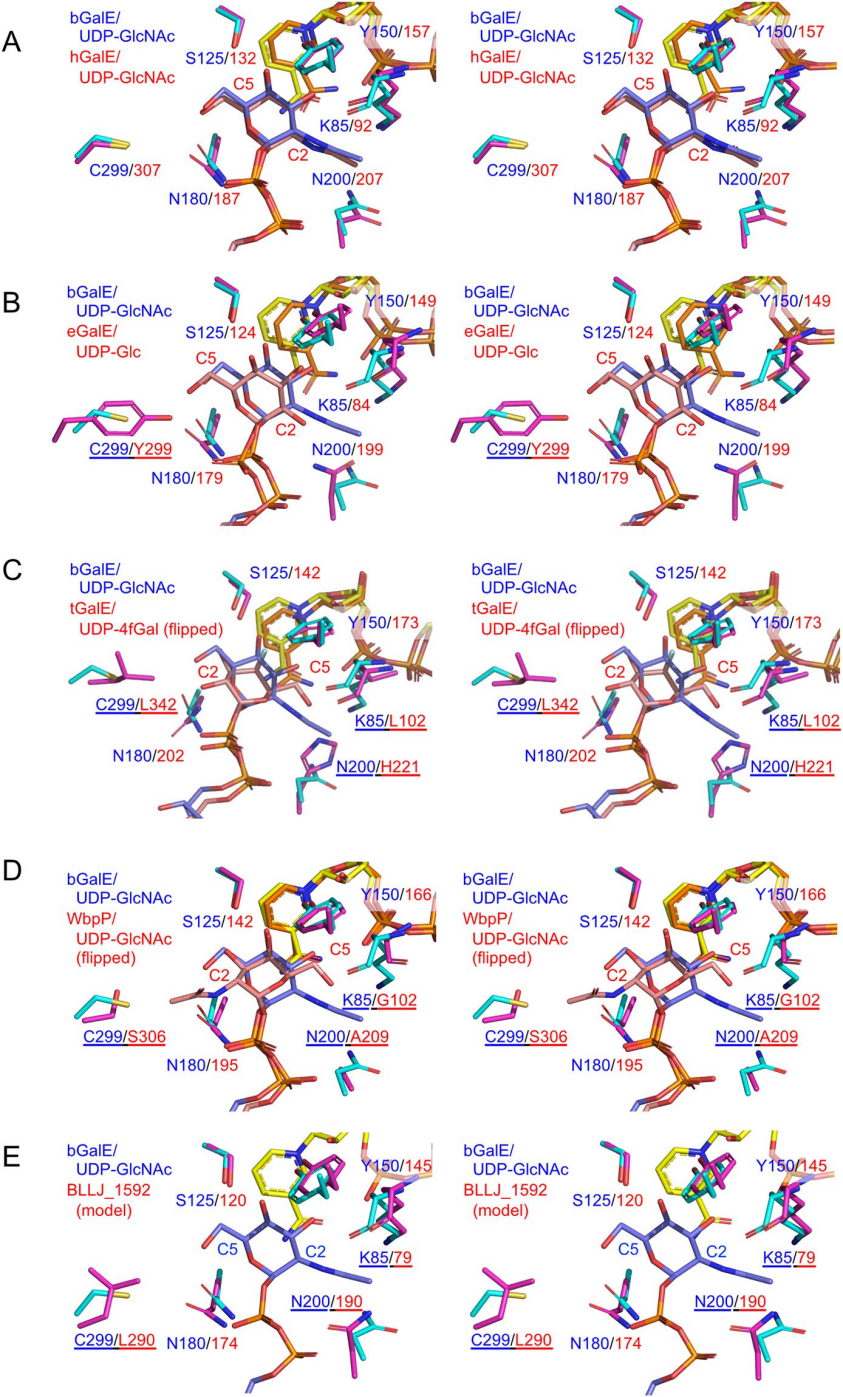
Stereoview of the UDP-sugar binding site in GalEs. Superimposition of bGalE + UDP-GlcNAc (cyan, blue, and yellow for protein, UDP-sugar, and NAD^+^) and other GalE enzymes (magenta, pink, and orange for protein, UDP-sugar, and NAD^+^) are shown. (**A**) hGalE + UDP-GlcNAc (PDB ID: 1HZJ chain B), (**B**) eGalE + UDP-Glc (PDB ID: 1XEL), (**C**) tGalE + UDP-4fGal (PDB ID: 2CNB chain A), (**D**) WbpP + UDP-GlcNAc (PDB ID: 1SB8), and (**E**) a homology model of BLLJ_1592.

Figure 6 shows superimpositions of the active site of bGalE + UDP-GlcNAc and representative enzymes in group 2 (hGalE), group 1b (eGalE), group 1a (tGalE), and group 3 (WbpP), presenting both the standard (bGalE + UDP-GlcNAc, hGalE + UDP-GlcNAc, and bGalE + UDP-Glc) and the flipped (tGalE + UDP-4-deoxy-4-fluoro-α-galactose (UDP-4fGal) and WbpP + UDP-GlcNAc) conformations of the sugar moiety. The UDP-GlcNAc complex structures of bGalE and hGalE (both in group 2) superimpose very well, and the residues forming the sugar binding site are completely conserved (Figs 5 and 6A). It is noteworthy that most UDP sugars observed in the crystal structures of GalE enzymes in groups 1 and 2 were in the standard conformation. For eGalE and tGalE in group 1, the flipped conformation structure was obtained only by using the S124A/Y149F double mutant^26^ or by using a substrate analog UDP-4fGal^39^. On the other hand, for a group 3 enzyme WbpP, the wild-type enzyme bound the natural substrate UDP-GlcNAc in the flipped conformation^32^.

**Figure 7:**
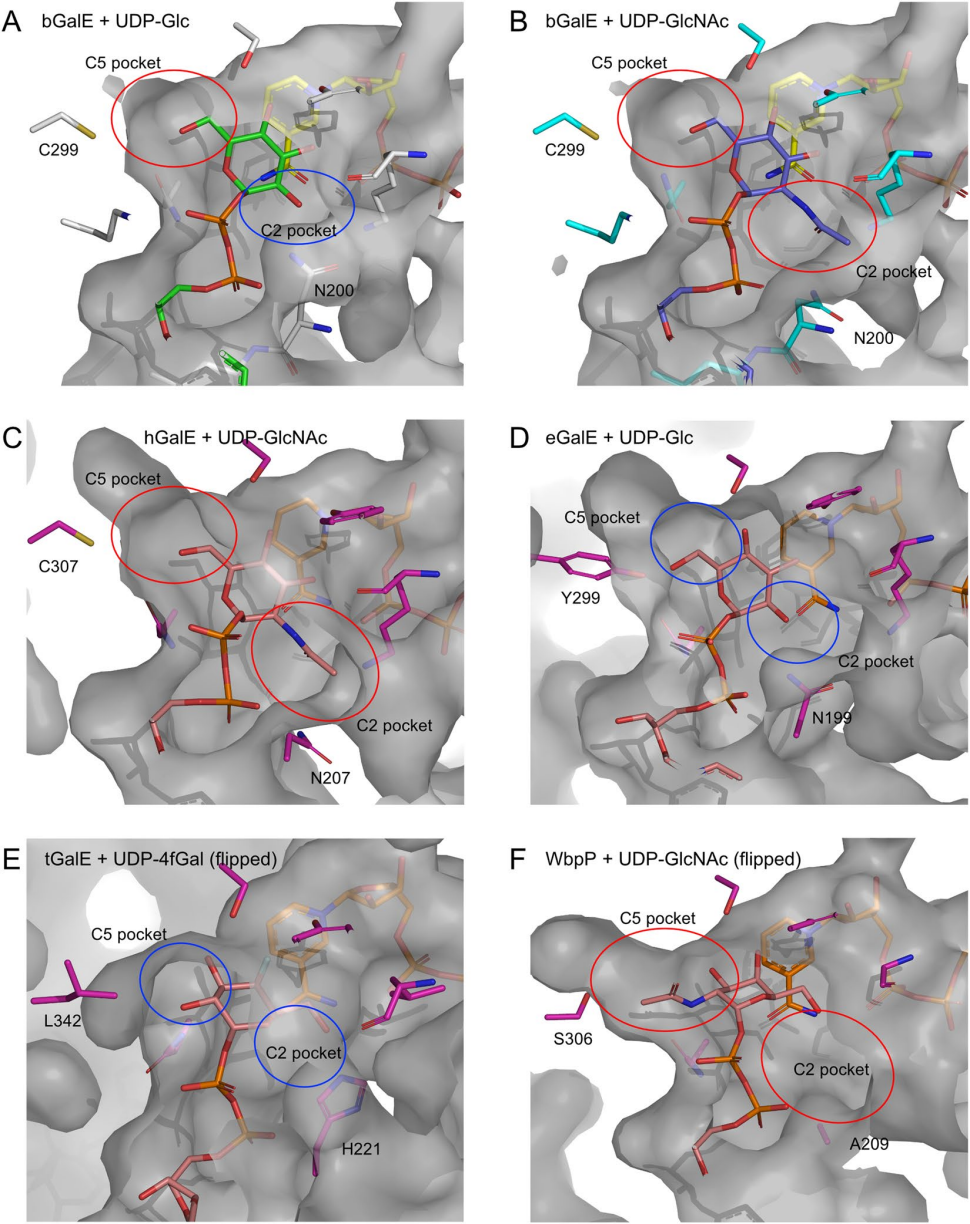
Surface presentation of the UDP-sugar binding site in GalEs. (**A**) bGalE + UDP-Glc, (**B**) bGalE + UDP-GlcNAc, (**C**) hGalE + UDP-GlcNAc, (**D**) eGalE + UDP-Glc (**E**) tGalE + UDP-4fGal, and (**F**) WbpP + UDP-GlcNAc are shown. Color codes and PDB IDs are as described in Figs 4 and 6. The C5 and C2 pockets are shown as red or blue circles for large or small pockets, respectively. The probe radius for surface calculation was 1.4 Å.

Ishiyama *et al*. proposed a conceptual model of the GalE active site surrounded by a hexagon-shaped box (six walls) and used it for discussion of the relationship between sequence (active site structure) and substrate specificity^32^. Here, we simplify the concept and focus on two pockets (walls in the original concept) responsible for the substrate specificity between UDP-Glc/Gal and UDP-GlcNAc/GalNAc. As shown in Fig. 7A,B, Asn200 and Cys299 form pockets for the groups connected to the C2 and C5 sugar ring atoms, respectively, in the “standard” conformation. Therefore, we hereafter designate these structural regions as C2 pocket and C5 pocket. These two pockets can discriminate the size of the C2 hydroxy or *N*-acetyl groups during the sugar group rotation in the catalytic cycle of the GalE reaction.

It has been shown that the size difference of the C5 pocket is the primary factor for the substrate specificity of GalEs^32^. Group 2 and 3 enzymes (bGalE, hGalE, and WbpP) have large C5 pocket with small Cys or Ser residue at this position whereas group 1 enzymes (eGalE and tGalE) have small C5 pocket with a bulky Tyr or Leu residue (Fig. 7). Although the crystal structure of eGalE Y299C mutant complexed with UDP-GlcNAc was reported, the sugar moiety was bound in a displaced nonproductive mode, and the distance between the C4 atom of GlcNAc and the C4 atom of nicotinamide ring was 9.4 Å^27^. The Y299C mutant of eGalE showed 5-fold reduction in the activity toward UDP-Gal, while it exhibited a 230-fold increase in activity toward UDP-GalNAc (Table 1). For hGalE, C307Y mutation resulted in significant loss of activity toward UDP-GalNAc, while the activity toward UDP-Gal was not affected (Table 1)^43^.

In the C2 pocket, the size and flexibility of the amino acid side chain contributed to accommodation of the *N*-acetyl group of the substrate. Similar to the case of bGalE observed in the present work, hGalE (group 2) adopts “swing in” and “swing out” conformations in the UDP-Glc and UDP-GlcNAc complex structures, respectively^29,30^. eGalE (group 1b) also has Asn199 residue at this position (Fig. 7D). However, in the crystal structure of the wild-type eGalE enzyme, the sugar moiety of UDP-GlcNAc was disordered^27^, suggesting limited flexibility of the Asn199 side chain. We further investigated the protein structures around the Asn residue and C2 pocket of the group 1b and 2 enzymes, but no significant structural feature that contribute to the flexibility difference between them was found (data not shown). The corresponding residues at the C2 pocket are large His221 in tGalE (group 1a specific for UDP-Glc) and small Ala209 in WbpP (group 3 specific for UDP-GlcNAc/GalNAc) (Fig. 7E,F). Therefore, the C2 pocket size is likely to contribute to the substrate specificity in these groups. In addition to the steric hindrance, a hydrogen-bonded solvent (water) network near Ala209 in WbpP is involved in the recognition of the *N*-acetyl group of the substrate^32^.

Three-dimensional structures of GalEs from a wide range of organisms are now available in the Protein Data Bank due to structural genomics projects and studies from several groups. Such efforts and advance in protein homology modeling have enabled more precise prediction of GalE specificities than the pioneering work by Ishiyama *et al*.^32^. We previously investigated biochemical characteristics of a paralog GalE enzyme from the same organism, *B. longum* JCM1217 (BLLJ_1592 corresponding to BL1671 of *B. longum* NCC2705)^44^, whose gene is not accompanied by other galactose metabolic genes. Although we did not measure the activity for UDP-GlcNAc/GalNAc, the specific activity of BLLJ_1592 for UDP-Gal (260 U/mg) was higher than that of bGalE (140 U/mg). Interestingly, BLLJ_1592 was suggested to be inhibited by GalNAc more severely than bGalE because the enzymatic GNB production using BLLJ_1592 under high GalNAc concentration (600 mM) was less efficient than that using bGalE. A phylogenetic tree analysis indicated that BLLJ_1592 is distantly located from any group (Fig. 2), and multiple amino acid sequence alignment could not predict its substrate specificity because of the low sequence homology around the C2 pocket-forming region (Fig. 5). We built a reliable homology model of BLLJ_1592 (GQME = 0.72 and QMEAN = −2.34) using the SWISS-MODEL server^45^ with an unpublished structure of GalE from *Burkholderia pseudomallei* (PDB ID: 3ENK, sequence identity = 42.3%) as a template. As shown in Fig. 6E, the C2 and C5 pocket of BLLJ_1592 are formed by Asn190 and Leu290, respectively. The inhibition by GalNAc suggests that the side chain of Asn190 is flexible and can accommodate the *N*-acetyl side chain. Therefore, BLLJ_1592 was predicted to have group 1-like specificity (low activity for UDP-GlcNAc/GalNAc) due to the large side chain of Leu290 in the C5 pocket.

### Concluding remarks

In this study, we revealed the structural basis for the broad substrate specificity of a bacterial enzyme that is required for efficient catabolism of HMO and mucin *O*-glycans. Interestingly, the structure of GalE from the human symbiont (*B. longum*) was more similar to that of its host animal (*H. sapiens*) compared to the enzyme from a gut microbe living in the same niche (*E. coli*). GalEs in group 1b and 2 are closely located in the phylogenetic tree (Fig. 2), and a small number of amino acid mutations in the active site (especially Cys or Tyr residue at the C5 pocket) can change the substrate specificity. Gal and GalNAc sugars are utilized by wide range of organisms from microbes to animals for various biological events such as breast feeding (lactose and HMO) and utilization of glycoproteins and glycoconjugates. To metabolize and utilize *galacto*-type sugars in relationship with other organisms (symbiosis or pathogenesis), organisms may be taking advantage of the GalE enzymes’ characteristics, such as the specificity being prone to change. Interestingly, hUXS1, which catalyzes the considerably different reaction from GalEs^40^, is located within the clade of group 3 GalEs in a phylogenetic tree (Fig. 2). The UDP-xylose synthase plays a key role in the glycosaminoglycan synthesis on the protein core of extracellular matrix proteoglycans of mammals. Moreover, a study using kinetic isotope effects reveled a more complicated reaction mechanism of UDP-D-apiose/UDP-D-xylose synthase, which is related to hUXS1. In addition to the NAD^+^-dependent oxidation and base catalysis by Tyr, the UDP-D-apiose/UDP-D-xylose synthase reaction involves decarboxylation, retro-aldol sugar ring opening, rearrangement, and ring contraction^46^. The unusually high catalytic potential of SDR superfamily enzymes cannot be estimated from the phylogenetic analysis alone but careful inspection based on three-dimensional protein structures is required.

## Methods

### Protein production and purification

A gene encoding C-terminally His6-tagged GalE (pET-30-*lnpD*, residues 1–340) was cloned from the genomic DNA of *B. longum* JCM1217 as previously described^15^. The expression plasmid was introduced into *E. coli* BL21 CodonPlus (DE3)-RIL (Stratagene, La Jolla, CA) for protein expression. The transformant was cultivated at 37 °C in Luria-Bertani medium containing 100 mg/L kanamycin and 20 mg/L chloramphenicol until the absorbance at 600 nm reached 0.6. Protein expression was induced by adding 0.1 mM isopropyl-β-D-thiogalactopyranoside to the medium. The culture was incubated for an additional 20 h at 25 °C. Expressed cells were harvested by centrifugation and suspended in 50 mM Tris-HCl (pH 7.0) and 0.1 mM phenylmethylsulfonyl fluoride. The cells were disrupted via sonication, and the supernatant was purified by sequential column chromatography. Ni-affinity chromatography was conducted using a HisTrap FF crude column (GE Healthcare, Fairfield, CT) with two steps of 20 mM and 250 mM imidazole in 50 mM Tris-HCl (pH 7.0). Gel-filtration chromatography was conducted using a Superdex 200 pg 16/60 column (GE Healthcare) equilibrated with 50 mM Tris-HCl (pH 7.0) and 150 mM NaCl at a flow rate of 1 mL/min. The purified protein concentrations were determined by a BCA protein assay kit (Thermo Fisher Scientific, Waltham, MA) with bovine serum albumin as the standard.

### Crystallography

Crystals of bGalE were grown at 4 °C using the sitting drop vapor diffusion method by mixing 1.0 µL of a 7 mg/ml protein solution with an equal volume of a reservoir solution containing 30% (v/v) PEG400, 0.2 M MgCl, and 0.1 M HEPES-NaOH (pH 7.5). Crystals grew in 2 weeks. Each complex was obtained by cocrystallisation with a ligand (10 mM UDP, 10 mM UDP-Gal, or 10 mM UDP-GlcNac). Crystals were flash-cooled at 100 K in a stream of nitrogen gas. The diffraction data sets were collected using synchrotron radiation at beamlines BL5A, BL17A, and AR-NW12A of the Photon Factory at the High Energy Accelerator Research Organization (KEK, Tsukuba, Japan). The bGalE crystals were long pillar-shaped, and we used helical scan mode for the data collection to reduce radiation damage. The data sets were processed using the HKL2000^47^ and XDS^48^. The initial phase was determined by the molecular replacement method using MOLREP^49^. Manual model rebuilding was achieved using Coot^50^. Crystallographic refinement was performed using Refmac5^51^. Babinet’s bulk solvent type scaling was applied, and TLS (Translation/Libration/Screw) refinement was not used. We used UDP-Gal for the soaking experiment but the resultant electron density map indicated that the protein was complexed with UDP-Glucose, suggesting that the catalytic conversion was occurred *in crystallo*. The refined crystal structures were validated using MolProbity (http://molprobity.biochem.duke.edu) server^52^. Molecular graphic images and amino acid sequence alignment were prepared using PyMOL (Schrödinger, LLC, New York, NY) and ESPript^53^.

### Accession codes

The coordinates and structure factors of UDP + NAD^+^, UDP-GlcNAc + NAD^+^, and UDP-Glc + NAD^+^ have been deposited in the Protein Data Bank under accession codes 6K0G, 6K0H, and 6K0I, respectively.
